# Cross-Family Mechanistic Analysis of Plant Alkaloids Against Neglected Arboviruses and Related RNA Viruses

**DOI:** 10.3390/molecules31152728

**Published:** 2026-08-06

**Authors:** Marcia Régis, Mario Fernando Sanchez Moreno, Hugo Germain, Natacha Mérindol, Isabel Desgagné-Penix

**Affiliations:** 1 Department of Biochemistry, Chemistry, Physics, and Forensic Science, Université du Québec à Trois-Rivières, Trois-Rivières, QC G9A 5H7, Canada; marcia.regis@uqtr.ca (M.R.); mario.fernando.sanchez.moreno@uqtr.ca (M.F.S.M.); hugo.germain@uqtr.ca (H.G.); 2 Plant Biology Research Group, Université du Québec à Trois-Rivières, Trois-Rivières, QC G9A 5H7, Canada

**Keywords:** neglected tropical diseases, flavivirus, mechanism of action, ribosome, structure-activity relationships, integrated stress response, broad-spectrum antivirals

## Abstract

Neglected arboviruses dengue (DENV), Zika (ZIKV), yellow fever (YFV), Japanese encephalitis (JEV), and chikungunya collectively affect hundreds of millions of people annually, yet no specific antiviral drug has been approved for any of them. Alkaloids, nitrogen-containing specialized metabolites produced by diverse plant families, have emerged as a promising source of broad-spectrum antiviral scaffolds. This review compiles and critically analyzes over 100 alkaloid-virus pairs across several RNA virus families, providing a comparative mechanistic analysis. Lycorine, narciclasine, emetine, and berbamine, among others, exhibit potent activity against phylogenetically distant viruses, with the most potent activities reported against flaviviruses (narciclasine: EC_50_ 0.02 µM against DENV, ZIKV, YFV, and JEV; pancratistatine: 0.0063 µM against ZIKV). Structure-activity analysis of multiple alkaloid classes identifies key pharmacophoric features, including the phenanthridine nucleus (lycorine derivatives) and the bis-benzylisoquinoline scaffold (tetrandrine, berbamine), as determinants of antiviral potency, selectivity, and broad-spectrum activity. Genetic resistance data in West Nile virus challenge the widely accepted model of lycorine as a direct nucleoside inhibitor, instead pointing toward the involvement of the membrane-associated NS4A-2K-NS4B replication complex, though direct validation remains limited for other flaviviruses. Converging structural, biochemical, and transcriptomic evidence suggests that ribosome-mediated translational stress may represent an additional host-directed antiviral mechanism for isoquinoline-type alkaloids, though the causal chain from ribosome binding to activation of the integrated stress response and to antiviral effect has not been established. The present analysis highlights that in vivo validation remains limited to a few alkaloid-virus pairs. Unbiased target deconvolution and formal testing of the ribosome/integrated stress response hypothesis stand out as essential research priorities.

## 1. Alkaloids to Fight Neglected Viral Diseases

Neglected tropical arboviruses, including dengue (DENV), Zika (ZIKV), yellow fever (YFV), Japanese encephalitis (JEV), and chikungunya (CHIKV), together threaten over half the world’s population, primarily in low- and middle-income countries [1]. Dengue alone accounts for an estimated 390 million infections per year, with 14.6 million cases notified to the WHO in 2024 and a population at risk projected to increase by 40% by 2035. While most patients recover within two weeks, all serotypes can induce symptoms ranging from high fever (40 °C/104 °F), severe headache, retro-orbital pain, muscle and joint pain, nausea, vomiting, swollen glands, and a skin rash, as well as life-threatening complications such as dengue hemorrhagic fever and dengue shock syndrome [2,3]. Reinfection significantly increases the risk of severe clinical manifestations, including abdominal pain, persistent vomiting, and mucosal bleeding. ZIKV is associated with congenital microcephaly. While most infections remain asymptomatic, clinical manifestations typically appear 3 to 14 days post-exposure, including rash, fever, conjunctivitis, and arthralgia, which may last up to a week. However, outbreaks over the last decade have highlighted severe neurological complications, such as Guillain-Barré syndrome in adults and microcephaly in newborns of infected pregnant women. YFV remains endemic in Africa and South America despite an effective vaccine, and JEV is the leading cause of viral encephalitis in Asia. CHIKV causes an acute febrile illness with severe polyarthralgia that can persist for months to years in 30–60% of patients, representing a significant long-term morbidity burden [4]. Major outbreaks since 2004 have spread the virus from East Africa to the Americas, the Caribbean, and Southeast Asia [5]. As with DENV and ZIKV, clinical management is limited to symptomatic support [2,6,7]. Fortunately, there have been significant advances in vaccines, with Qdenga (TAK-003) approved in over 40 countries for DENV as of 2025 [8], and the single-dose Butantan-DV vaccine demonstrating 65.0% overall efficacy and 80.5% protection against severe disease over 5 years in a phase 3 trial, regardless of baseline serostatus [9]. Regarding CHIKV, Ixchiq (VLA1553) received FDA approval in 2023, although post-marketing safety monitoring is ongoing in 2026 [10]. On the antiviral front, mosnodenvir (JNJ-1802), a first-in-class pan-serotype DENV inhibitor targeting the NS3-NS4B interaction, demonstrated prophylactic efficacy in a controlled human infection model, but its clinical development was discontinued in 2024 for reasons unrelated to safety [11,12]. No antiviral drug for any neglected arbovirus has reached final approval as of June 2026, underscoring the continued need for novel therapeutic approaches.

**Figure 1:**
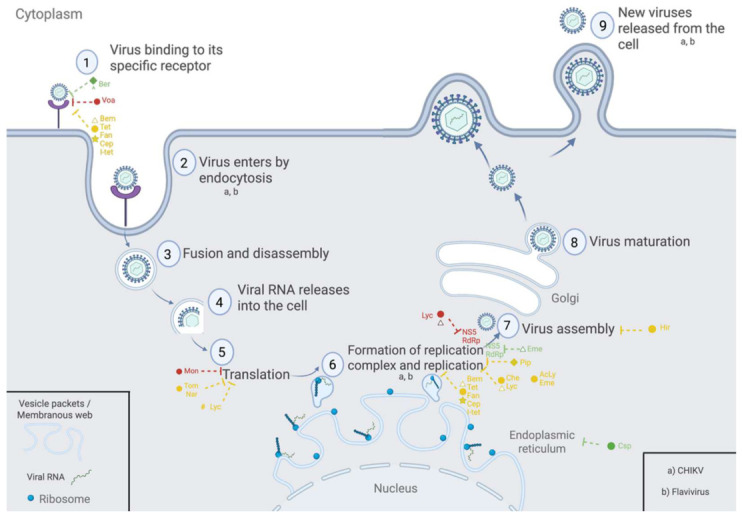
Replication cycle of the *Flaviviridae* family and CHIKV, with alkaloid targets indicated. (1–2) Attachment to specific receptors (e.g., DC-SIGN receptor (CD209) for DENV, AXL receptor for ZIKV; CD81/claudin-1 for HCV) and entry via endocytosis. (3–4) Endosomal fusion and release of the (+) RNA genome. (5–6) Translation of the polyprotein and assembly of the non-structural (NS) protein complex within specialized ER-derived organelles (vesicle packets or membranous web), where genomic replication and production of RNAs occur. (7–8) Viral assembly and maturation through the Golgi apparatus via cleavage by the host peptidase furin. (9) Egress of mature virions. Specific symbols have been used to identify viruses. • DENV, Δ ZIKV, ♦ HCV, ★ JEV, **^** YFV, **#** CHIKV. Target confidence is indicated by color: green indicates molecularly validated targets (crystallographic, biochemical enzyme assay, or genetic resistance data), yellow indicates step-level assignments (time-of-addition or replicon experiments without identified molecular target), and red indicates proposed mechanisms based on indirect evidence or extrapolation from other viral systems. Berberine (Ber ♦, **^**), voacangine (Voa •), berbamine (Bem •, Δ, ★), tetrandrine (Tet •, Δ, ★), fangchinoline (Fan •, Δ, ★) cepharanthine (Cep •, Δ, ★), and iso-tetrandrine (I-tet •, Δ, ★), target the early stages of infection, specifically viral entry and endosomal trafficking. Montanine (Mon •), Narciclasine (Nar •), tomatidine (Tom •) and lycorine (Lyc **#**) are proposed to interfere with translation and polyprotein processing. Lycorine (Lyc •, Δ), acetyllycorine (AcLyc •), cherylline (Che •, Δ), piperine (Pip •), emetine (Eme ●, Δ) and also berbamine (Bem •, Δ, ★), tetrandrine (Tet •, Δ, ★), fangchinoline (Fan •, Δ, ★), cepharanthine (Cep •, Δ, ★) and iso-tetrandrine (I-tet •, Δ, ★) target the replication phase in most studies. Hirsutine (Hir •) has been identified as an inhibitor of viral assembly. Castanospermine (Csp •) acts on the final maturation of viral particles by inhibiting host α-glucosidases within the endoplasmic reticulum (ER). References are available in Table 2. Although CHIKV (a) belongs to the *Togaviridae* (genus Alphavirus), its replication cycle shares key features with flaviviruses (b), including receptor-mediated endocytosis, cytoplasmic replication within membrane-associated complexes, and budding of enveloped particles; CHIKV alkaloid data are therefore compiled alongside flaviviruses in Table 3. Created with BioRender.com.

Dengue and chikungunya are formally recognized by the WHO as neglected tropical diseases (NTDs), while ZIKV, YFV, and JEV are managed under the same Global Arbovirus Initiative framework. The co-circulation of DENV, ZIKV, and CHIKV through shared *Aedes* mosquitoes creates a compounding public health challenge in endemic regions, where differential diagnosis is often unavailable at the point of care. These RNA viruses, despite their phylogenetic diversity, share a common replication logic. Infection proceeds through sequential steps, including receptor binding and endosomal entry, translation of the viral genome by host ribosomes in the cytoplasm, proteolytic processing of the polyprotein, assembly of membrane-associated replication organelles, RNA synthesis, and virion assembly and release (Figure 1). Each of these steps constitutes a potential therapeutic target. For some related viruses, this logic has been successfully exploited, e.g., DAAs targeting the hepatitis C virus (HCV) NS5B polymerase or NS3 protease have transformed patient outcomes [13], and nirmatrelvir inhibits the SARS-CoV-2 main protease. However, for the neglected arboviruses, no drug targeting any of these steps has yet reached final approval.

Alkaloids, nitrogen-containing specialized metabolites produced across plants, fungi, bacteria, and animals, have long been recognized for their pharmacological properties. They are particularly abundant in medicinal plants, where they exhibit the greatest structural diversity (Table 1) [14,15]. In their natural hosts, they play a role in defense against predators and pathogens [16,17] and are distributed throughout various tissues, where they may accumulate at concentrations ranging from 1 to 15% of plant dry weight [18,19]. Over 27,600 alkaloids have been characterized across major structural classes. In medicinal chemistry, these molecules are classified based on their underlying chemical scaffolds, which dictate both their biosynthetic origins and their spatial interactions with biological targets. These primary classes include indole and monoterpene indole scaffolds, isoquinolines (IQ), benzylisoquinolines (BIQ), bis-benzylisoquinolines (bis-BIQ), phenanthridines, tropanes, piperidines, iminosugars, and steroidal frameworks [20,21,22,23,24]. Several have become cornerstones of modern medicine, such as morphine (analgesic), quinine (antimalarial), emetine (antiamoebic), vincristine (antineoplastic), and galanthamine (anti-cholinesterase), demonstrating their potential as clinically viable drugs. Despite the extensive in vitro antiviral activity, no alkaloid or alkaloid-derived compound has been approved as an antiviral drug for any indication. The only candidate to reach clinical trials for a neglected arbovirus, celgosivir, a castanospermine prodrug targeting host α-glucosidases, failed to demonstrate efficacy in phase II despite a favorable safety profile [25], underscoring the need for a deeper mechanistic understanding before advancing further candidates.

**Table 1:** Medicinal plant families producing antiviral alkaloids.

Plant Family	Antiviral Alkaloid	Geographical Distribution	Domains of Application	Ref.
*Solanaceae* (Tropane)	Atropine	Cosmopolitan, especially tropical America	Food, Medicinal, Ornamental	[16]
*Rutaceae* (Isoquinoline/Benzo[c]phenanthridine)	Octopamine, 5,6-Dihydro-6-methoxynitidine	Tropical and subtropical zones	Food, Medicinal Cosmetics, Ornamental	[26]
*Rubiaceae* (Isoquinoline/Ipecac and Monoterpene indole)	Emetine, hirsutine	Tropical and subtropical zones	Medicinal (antiviral)	[27,28]
*Piperaceae* (Piperidine)	Piperine	Tropical zones, Latin America	Medicinal (antiviral)	[16]
*Menispermaceae* (Bis-benzylisoquinoline/bis-BIQ)	Cepharanthine, fangchinoline, tetrandrine	Warm temperate to tropical zones	Industrial, Medicinal (antiviral)	[28]
*Lauraceae* (Aporphine)	Hernandonine Laurolitsine	Pantropical to warm temperate	Medicinal (Antiviral, antitumor), Culinary	[29]
*Liliaceae* (Steroidal)	Cyclopamine	Cosmopolitan	Food, Medicinal, Ornamental	[23]
*Fabaceae* (Indolizidine/Quinolizidine)	7-oxohernangerine	Cosmopolitan	Food, Medicinal, Ornamental	[20]
*Cephalotaxaceae* (Cephalotaxine/Cephalotaxane)	Lindechurine A	Asia, North America	Medicinal (antiviral)	[30]
*Berberidaceae* (Isoquinoline: protoberberine & bis-BIQ)	Berberine, Berbamine	Subtropical zones	Food, Medicinal (Antiviral, antimycobacterial, anti-inflammatory), Homeopathy	[31]
*Apocynaceae* (Monoterpene indole/Phenanthroindolizidine)	Ajmalicine, vocangine, 18-Methoxycoronaridine, tylophorine	Tropical, subtropical zone	Medicinal, Ornamental	[27]
*Amaryllidaceae* (Amaryllidoideae: Lycorine, Crinine, Haemanthamine, Narciclasine, Cherylline types)	Cherylline, crinamine, haemanthamine, haemanthidine, lycorine, Narciclasine, pancracine, pancratistatine	Tropical, subtropical, temperate zones	Medicinal (antitumoral, antiviral, etc.), Ornamental	[17,32,33,34,35]

Ref.: Reference.

**Table 2:** Reported antiviral activity and mechanism of action of diverse alkaloid families against Dengue and Zika viruses.

	Alkaloid	EC_50_ (μM)	CC_50_ (μM)	SI	Target	Ref.
**DENV**						
*Amaryllidaceae*	Acetyllycorine	0.4	>300	>750	Replication	[3]
	Lycorine	0.48–0.59	>10	>17	Replication; RNA synthesis	[36,37]
	1-O-acetyl-2-oxolycorine	1.8	>300	>167	n.e.	[38]
	2-Oxolycorine	0.5	>300	>600	n.e.	[3]
	Lycoricidine monoacetate	5.15	>143	>28	n.e.	[39]
	Lycoricidine triacetate	>17.17	>114	>7		
	(+)-*trans*-dihydronarciclasine	0.51	>159	>311		
	7-Deoxyisonarciclasine	28.99	>181	>6		
	7-Deoxy-*trans*-dihydronarciclasine	1.17	>170	>145		
	*cis*-dihydronarciclasine	7.11	>159	>22		
	7-Deoxypancratistatin	2.15	>170	>79		
	Pseudolycorine	1.35	25.26	19	n.e.	[39]
	Pancracine	0.36	25.93	72	n.e.	[40]
	Haemanthamine	0.34	2.19	6		
	Haemanthidine	0.48	16.80	35		
	Pretazettine	0.8	11.45	14		
	Pancratistatine	0.19	1.71	9.0		
	Vittatine	15.53	n.d.	n.d.		
	11-Hydroxyvittatine	3.92	113.06	29		
	Cherylline	8.8	>100	>11	Replication	[36,41]
	Narciclasine	0.02	0.09	5	n.e.	[42]
	Narciclasine-4-O-β-D-xylopyranoside	7.9	39.3	5		
	Montanine	0.06–0.26	2.67	10–44	Translation	[43] ^#^
*Berberidaceae*	Berbamine	0.54	76.35	141	Entry and replication (host TRPML channels and endosomal trafficking)	[44]
	Berberine	0.7	205.1	293	infectious particle formation; host p38 MAPK	[45]
*Fabaceae*	Castanospermine	1	>5000	>5000	Incorporation of viral protein (prM-E) and C.	[46]
*Menispermaceae*	Cepharanthine	0.06	12.31	205	Entry and replication (host TRPML channels and endosomal trafficking)	[44]
	Fangchinoline	0.06	52.83	881		
	Tetrandrine	0.42	9.50	23		
	Iso-tetrandrine	0.27	18.60	69		
*Rubiaceae*	Emetine	0.12	>10	>83	Replication	[47]
	Hirsutine	0.57	>50	>88	Assembly	[48]
*Solanaceae*	Tomatidine	0.82	80.2	98	Synthesis of viral protein E	[49]
**ZIKV**						
*Amaryllidaceae*	Cherylline	20.30	>100	>5	Replication	[36]
	Lycorine	0.41	>10	>24		
	Narciclasine	0.02	0.12	6.0	n.e.	[42]
	Narciclasine-4-O-β-D-xylopyranoside	7.90	51.58	7		
	Pretazettine	1.90	>100	>53		
	(+)-*trans*-dihydronarciclasine	0.031	0.18	6	n.e.	[33]
	Pancratistatine	0.0063	>150	>23,809	n.e.	[39]
*Berberidaceae*	Berbamine	1.90	76.35	40	Entry (host TRPML channels and endosomal trafficking) and replication	[44]
	Berberine	0.2	15.3	77	Infectious particle formation, host p38 MAPK	[45]
*Menispermaceae*	Cepharanthine	0.37	18.60	50	Replication (host cells)	[44]
	Fangchinoline	4.77	9.50	2		
	Tetrandrine	1.29	12.31	10		
	Iso-tetrandrine	2.11	51.83	25		
*Rubiaceae*	Emetine	^ IC_50_ 0.121	n.d.	n.d.	NS5 RdRp	[50]

^#^ This study is a preprint (not peer-reviewed). Ref.: Reference. EC_50_: concentration of 50% inhibition of infection, CC_50_: concentration of 50% cytotoxicity. ^: represents biochemical IC50, measured through a cell-free assay directly evaluating an enzyme inhibition. SI: selectivity index (CC_50_/EC_50_ or IC_50_). > indicates that the maximum nontoxic concentration tested did not reach the 50% cytotoxicity threshold; the corresponding SI values represent minimal calculated selectivity. ± standard deviation of the mean. n.d.: Not determined. n.e.: not elucidated. TRPML: Transient Receptor Potential Mucolipin. MAPK: Mitogen-Activated Protein Kinase. RdRp: RNA-dependent RNA polymerase.

**Table 3:** Reported antiviral activity and mechanism of action of diverse alkaloid families against various flaviviruses and CHIKV.

Virus	Alkaloid	EC_50_ (μM)	CC_50_ (μM)	SI	Target	Ref.
JEV	*cis*-dihydronarciclasine	41.06	202.09	5	n.e.	[39]
	Lycorine	1.04	9.40	9		
	Narciclasine	* IC_50_ 0.026	0.101	4		
	7-Deoxypancratistatin	1.55	9.05	6		
	Cepharanthine	0.5063	18.60	37	Replication (host cells)	[44]
	Fangchinoline	3.870	9.50	2		
	Tetrandrine	5.172	12.31	2		
	Iso-tetrandrine	5.751	51.83	9		
	Berbamine	2.54	76.35	30		
YFV	*cis*-dihydronarciclasine	31.04	206.94	7	n.e.	[39]
	Lycorine	0.79	7.10	9		
	Narciclasine	* IC_50_ 0.0195	120	6		
	7-Deoxypancratistatin	1.29	8.41	7		
	Berbamine	2.541	76.35	30	Entry and replication (host TRPML channels and endosomal trafficking)	[44]
AHFV	Lycorine	1.7	16.2	10	n.e.	[39]
WNV	Lycorine	0.23	24	104	Replication complex (peptide 2K)	[38]
CHIKV	Berberine	1.8 ± 0.5	>100	>56	Protein synthesis	[51]
	Harringtonine	0.24	n.d.	n.d.	Translation	[30]
	Lycorine	~1 * IC_50_ 0.319	n.d. >50	n.d. >156	Translation Post-entry and RNA synthesis	[37,52]
	Tomatidine	1.3	80.2	62	Viral protein expression	[53]
	^#^ Emetine	0.01	9.67	967	Nsp2 helicase	[54]

^#^ This study is a preprint (not peer-reviewed). Ref.: Reference. EC_50_: concentration of 50% inhibition of infection. *: represents IC50 values determined using a cell-based phenotypic assay. CC_50_: concentration of 50% cytotoxicity. SI: selectivity index (CC_50_/EC_50_ or IC_50_). > indicates that the maximum nontoxic concentration tested did not reach the 50% cytotoxicity threshold; the corresponding SI values represent minimal calculated selectivity. ± standard deviation of the mean. n.d.: Not determined. n.e.: Not elucidated. TRPML: Transient Receptor Potential Mucolipin. JEV: Japanese Encephalitis Virus. YFV: Yellow Fever Virus. AHFV: Alkhurma Hemorrhagic Fever Virus. WNV: West Nile Virus. CHIKV: Chikungunya Virus.

Antiviral activity of plant-derived alkaloids has been increasingly documented since the early 1990s [39], and multiple recent reviews have cataloged their activity [16,55,56]. While these reviews provide valuable compilations of antiviral alkaloids organized by virus or alkaloid class, the present work takes a complementary approach by discussing the presented evidence by molecular target, enabling cross-family comparisons and a critical assessment of the strength of each mechanistic claim.

Plant alkaloids are distributed across a broad evolutionary range of families (Table 1), notably within large families such as *Solanaceae*, *Fabaceae*, and *Rubiaceae*. Tropical lineages, including *Rutaceae*, *Apocynaceae*, and *Piperaceae*, are also identified as significant sources of structural diversity. These botanical families often exhibit highly specialized biosynthetic pathways that yield distinct structural scaffolds. For instance, the *Menispermaceae* comprise numerous species known for bis-benzylisoquinoline alkaloids, and the *Amaryllidoideae* (a subfamily of *Amaryllidaceae*) are an exceptionally prolific group; approximately 20% of their species produce specialized alkaloids belonging to unique isoquinoline-derived structural types (such as lycorine, crinine, haemanthamine, and narciclasine cores) [32]. Notably, these alkaloid-producing families are largely endemic to tropical and subtropical regions where arboviral transmission is most intense (Table 1), suggesting that traditional medicinal plant use in these communities may yield leads for antiviral drug discovery.

Non-arboviral RNA viruses (HCV, coronaviruses, influenza, HIV-1, RSV, EBOV) are included where mechanistic data are available to provide a comparative framework, as target identification for these better-resourced pathogens can inform and contextualize the more limited evidence available for neglected arboviruses. Data on DNA viruses were excluded and are summarized elsewhere [55,56]. The analysis is discussed by molecular target, encompassing viral entry, polymerase activity, protease-dependent processing, ribosome binding, and host innate immune modulation. The strengths of the evidence for target claims are discussed, and the most promising research opportunities are highlighted.

## 2. Research Methodology

Relevant literature was identified by searching PubMed, Scopus, Web of Science, ScienceDirect, and Google Scholar, with searches limited to publications up to June 2026. Primary searches combined broad terms (“alkaloid” AND “antiviral”, “alkaloid” AND “RNA virus”) with virus-specific queries (“alkaloid” AND “dengue”/“Zika”/“coronavirus”/“flavivirus”/“chikungunya”/“neglected virus”/“tropical virus”/“influenza”/“HCV”/“HIV”). Complementary searches combined plant family names (such as “*Amaryllidaceae*”, “*Menispermaceae*”, “*Apocynaceae*”, “*Fabaceae*”) with “antiviral” to capture alkaloids not indexed under individual compound names. When an alkaloid with documented antiviral activity was identified, a targeted secondary search using the compound name (e.g., “lycorine”, “berberine”, “emetine”) was performed to retrieve mechanistic data across additional viral contexts and beyond. Inclusion criteria required: (1) isolated, pure, plant-derived alkaloids or their semi-synthetic derivatives; (2) antiviral activity against RNA viruses demonstrated in cell-based or biochemical assays with quantitative endpoints (EC_50_, IC_50_, or equivalent); and (3) characterized or proposed molecular mechanisms of action. Exclusion criteria were: (1) studies focusing exclusively on DNA viruses; (2) crude plant extracts with undefined active principles; and (3) purely computational studies without experimental validation, which were cited for mechanistic context but not compiled in the activity tables. One preprint with significant mechanistic data [43] was included and is clearly identified as such throughout the manuscript. Approximately 120 compound-virus pairs extracted from the selected literature were categorized by molecular target and examined across viral families to identify conserved antiviral mechanisms. This work constitutes a critical narrative review.

## 3. Viral Targets of Alkaloids: Cross-Family Analysis

RNA viruses are a major contributor to the emergence of new infectious diseases [57]. Their rapid evolution and mutation rate make them challenging pathogens to eradicate, requiring specific vaccination and antiviral strategies [58]. The viruses covered in this review span a broad spectrum of replication strategies, tissue tropisms, and clinical impacts. Members of the *Flaviviridae* include neglected arboviruses transmitted by *Aedes* mosquitoes (DENV, ZIKV, YFV, JEV) and the blood-borne HCV. *Coronaviridae* range from endemic seasonal pathogens (e.g., Human coronavirus (HCoV)-OC43) to epidemic and pandemic viruses (e.g., severe acute respiratory syndrome (SARS)-CoV, Middle East respiratory syndrome (MERS)-CoV, and SARS-CoV-2). *Orthomyxoviridae* (e.g., influenza virus) cause recurrent seasonal epidemics with pandemic potential. Additional viruses (CHIKV, Ebola virus (EBOV), human immunodeficiency virus (HIV-1), respiratory syncytial virus (RSV), parainfluenza virus (HPIV)-3) are included where alkaloid data are available.

**Figure 2:**
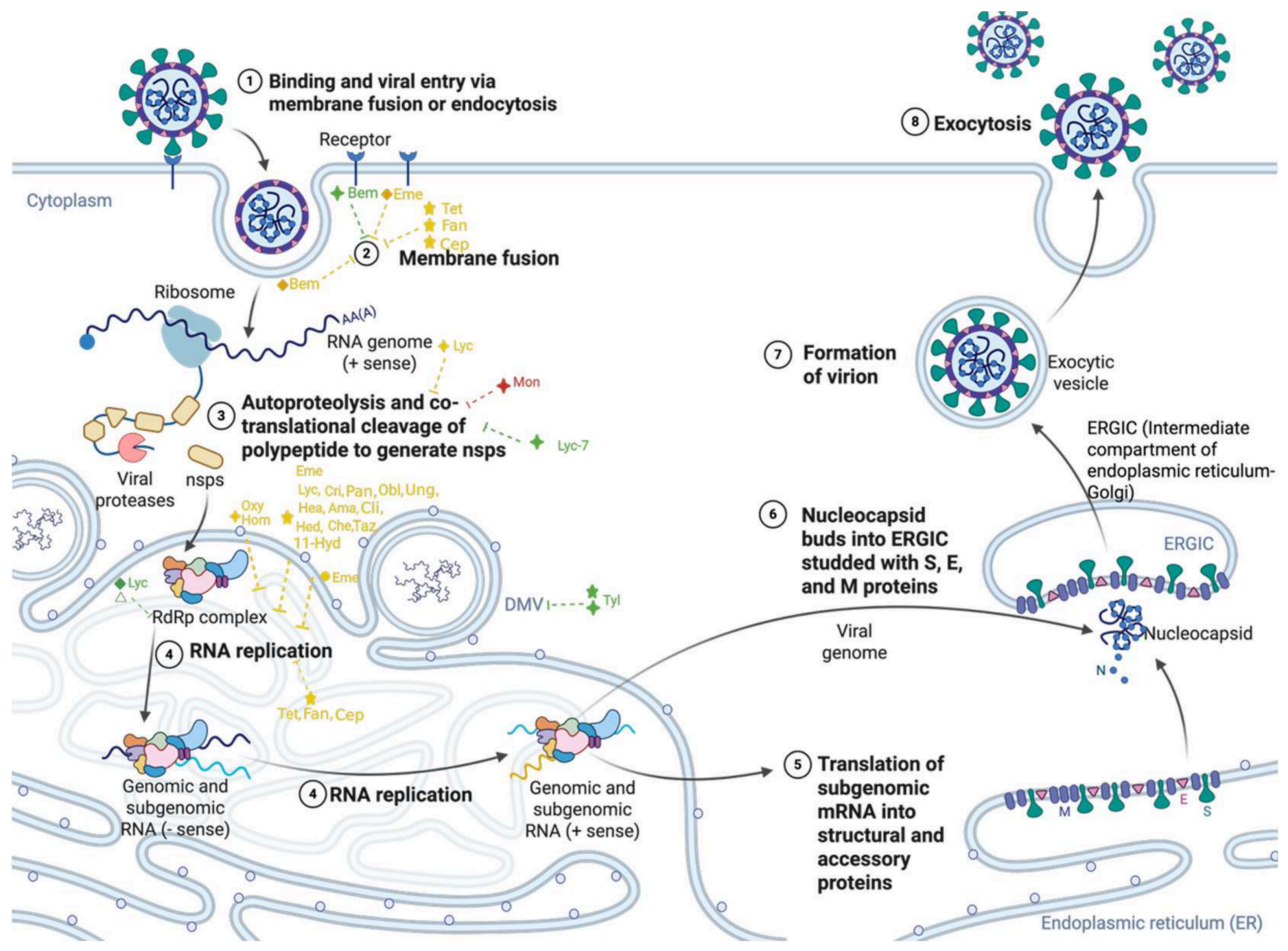
Replication cycle of the *Coronaviridae* family, with alkaloid targets indicated. (1) Attachment to host receptors, such as angiotensin-converting enzyme 2 (ACE2) for SARS-CoV-2 or 9-O-acetylated sialic acids for HCoV-OC43, which involves the hemagglutinin-esterase (HE) protein. (2) Entry and release of the genomic RNA by endocytosis. (3) Polyprotein translation (pp1a/pp1ab) and autocleavage into Nsps. (4) Replication-transcription complex (RTC) assembly and RNA replication/transcription within double-membrane vesicles (DMVs). (5) Translation of subgenomic mRNAs into structural and accessory proteins. (6–7) Viral assembly in the endoplasmic reticulum-Golgi intermediate compartment (ERGIC) and maturation. (8) Release via exocytosis, where the HE protein facilitates HCoV-OC43 detachment. Specific symbols in red are used to denote the antiviral activity and molecular targets of the discussed alkaloids. ★ HCoV-OC43 and 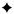 SARS-CoV-2, Δ SARS-CoV, ♦ MERS-CoV, • HCoV-NL63. Target confidence is indicated by color: green indicates molecularly validated targets (crystallographic, biochemical enzyme assay, or genetic resistance data), yellow indicates step-level assignments (time-of-addition or replicon experiments without identified molecular target), and red indicates proposed mechanisms based on indirect evidence or extrapolation from other viral systems. Berberine (Ber 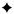,♦) directly targets membrane fusion, while Emetine (Eme ♦) and Bis-benzylisoquinolines such as tetrandrine (Tet ★), fangchinoline (Fan ★), and cepharanthine (Cep ★) block the early infection process. In the host cytosol, translational elongation is modulated at the ribosomal PTC by Lycorine derivative compound 7 (Lyc-7 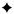), which stabilizes host ZAP-S to disrupt essential -1 programmed ribosomal frameshifting (-1PRF). Lycorine (Lyc 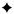) also acts at this level as a potent inhibitor of ribosomal elongation. Conversely, translational modulation at the ribosomal level by montanine (Mon 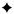) may trigger ribosome collisions. Lycorine (Lyc ♦ Δ) is shown to inhibit RdRp. Emetine (Eme ★), homoharringtonine (Hom 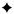), oxysophoridine (Oxy 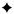) and tylophorine (Tyl ★,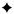) block the replication stage. A diverse group of *Amaryllidaceae* alkaloids, including lycorine (Lyc ★), haemanthamine (Hae ★), haemanthidine (Hed ★), crinamine (Cri ★), amarbellisine (Ama ★), cherylline (Che ★), pancracine (Pan ★), ungeremine (Ung ★), clivimine (Cli ★), tazettine (Taz ★), obliquine (Obl ★) and 11-hydroxyvittatine (11-hyd ★) interfere with post-entry mechanisms within 4 to 8 h post-infection. Adapted from BioRender.com, https://doi.org/10.1016/j.it.2020.10.004 and https://doi.org/10.1038/s41579-020-00468-6. Detailed EC_50_ values and mechanistic evidence for each compound are provided in Table 5.

Despite their diversity, these viruses share common vulnerabilities that alkaloids may exploit, including dependence on host translational machinery, membrane-associated replication complexes, and the encoding of conserved enzymes, such as polymerases and viral proteases (Figure 1 and Figure 2). Antiviral alkaloids focus on several distinct molecular targets. Table 2, Table 3, Table 4, Table 5 and Table 6 compile the antiviral activities of alkaloids reported against DENV and ZIKV (Table 2), other flaviviruses, CHIKV (Table 3), HCV (Table 4), coronaviruses (Table 5), influenza virus, HIV-1, and other relevant viruses (Table 6). There was heterogeneity in endpoints (e.g., EC_50_, IC_50_) when compiling antiviral data from studies spanning several decades and multiple virus families. Phenotypic EC_50_ values reflect a compound’s ability to inhibit viral replication in cell culture, while biochemical IC_50_ values measure direct inhibition of a purified viral or host enzyme. These distinct metrics are indicated across tables. Differences in absolute values across studies may be attributable to variations in cell lines, viral strains, and assay formats. For this reason, the present review focuses on mechanistic patterns and target assignments rather than on quantitative ranking of compound potencies across studies.

### 3.1. Viral Entry Inhibition

Entry inhibition is a pharmacologically diverse mechanism reported for antiviral alkaloids, spanning at least three distinct molecular steps. Bis-BIQ alkaloids are well-characterized entry inhibitors. Tetrandrine, fangchinoline, and cepharanthine inhibit HCoV-OC43 infection of human lung cells, likely by disrupting endolysosomes (Table 5 and Figure 2) [64]. Berbamine acts at a different step, binding to the post-fusion core of the SARS-CoV-2 Spike S2 subunit and blocking membrane fusion [67]. Since acidification is also required for pH-triggered fusion of flaviviruses in late endosomes, recent studies have confirmed that all five bis-BIQ tested (berbamine, tetrandrine, iso-tetrandrine, fangchinoline, and cepharanthine) inhibit ZIKV, DENV, and JEV (Table 2 and Table 3) infection by blocking both entry and replication [44]. The entry-inhibition mechanism involves blockade of Transient Receptor Potential-Mucolipin (TRPML) channels, lysosomal pH alkalization, and reduced expression of the ZIKV receptor, NCAM1 [85,86]. While these results suggest that the dimeric bis-BIQ scaffold may favor interaction with endolysosomal targets, the breadth of this activity across additional viruses and experimental systems remains to be established. HIV-1 enters cells via fusion rather than endocytosis, yet this step can also be blocked by some alkaloids, such as aloperine, a quinolizidine-type alkaloid (Table 6) [80].

The IQ alkaloid berberine displays a less conserved antiviral mechanism. Against HCV, Hung et al. (Table 4) [60] demonstrated that berberine binds the E2 glycoprotein, blocking entry with a classical receptor-level mechanism. However, during DENV and ZIKV infections, berberine does not affect entry (Table 2) [45]. Likewise, emetine exhibits a virus-dependent pattern, inhibiting MERS-CoV and EBOV entry (Table 5 and Table 6), but targeting later steps of ZIKV replication [50,65]. This underscores that mechanistic data from one virus cannot directly be extrapolated to another. A dual-mechanism profile, entry blockade for one virus, replication inhibition for another, is potentially advantageous for a broad-spectrum antiviral, as it reduces the likelihood that resistance at a single target will abolish efficacy across viral families.

Voacangine, a monoterpenoid indole alkaloid (MIA), and its derivatives were virucidal for some strains of DENV and CHIKV with no post-treatment effect (Table 2 and Table 3) [87,88]. Interestingly, to date, several *Amaryllidaceae* alkaloids have been tested in time-of-addition or entry-specific assays, including lycorine against influenza H5N1 (Table 6) [89], DENV (Table 2) [36], CHIKV (Table 3) [37,52]; and seven alkaloids against HCoV-OC43 (Table 5) [62] have shown post-entry activity. While this does not exclude the possibility that untested compounds or virus-alkaloid pairs may involve entry inhibition, the consistency of these findings across multiple viral families and diverse alkaloid scaffolds supports a shared post-entry activity for this alkaloid class.

### 3.2. RNA-Dependent RNA Polymerase Inhibition

Most RNA virus replication requires the expression of an RNA-dependent RNA polymerase (RdRp) that can replicate its genome (Figure 1 and Figure 2). Retroviruses, such as HIV-1, use a reverse transcriptase that allows their genome to transition to a DNA provirus. As specifically encoded by the virus, these polymerases are a frequently reported target for antiviral alkaloids, but the strength of evidence varies dramatically across viral families and compounds. For lycorine, the most compelling evidence for polymerase inhibition comes from the coronavirus field (Table 5). Jin et al. [72] demonstrated that lycorine directly inhibits RdRp activity with an IC_50_ of 1.4 µM in a cell-based reporter assay, a value concordant with its cellular EC_50_ and more potent than remdesivir (IC_50_ 6.3 µM) in the same system. However, because the assay is cell-based, indirect effects, such as reduced RdRp translation, cannot be excluded, and the system does not recapitulate the full viral replication cycle. As a result, it remains uncertain whether RdRp inhibition is the primary antiviral mechanism or a correlative observation. Purified enzyme assays or subgenomic replicon systems would be needed to resolve this question.

Lycorine maintains submicromolar activity against all tested flaviviruses and has demonstrated significant in vivo efficacy, with an 83% improvement in survival in ZIKV-infected mice [90]. However, its possible activity as a direct NS5 RdRp inhibitor is considerably weaker than widely assumed. Using subgenomic replicons, our results demonstrated that lycorine and cherylline inhibit DENV RNA replication [36]. In addition, Agrawal et al. (2024) confirmed that lycorine reduces total and negative-strand RNA synthesis across all four DENV serotypes and CHIKV [37]. Molecular docking placed lycorine near the catalytic sites of both DENV and CHIKV RdRps [37], but these remain computational predictions. Indeed, while biochemical assays in the literature report an IC_50_ for the purified ZIKV NS5 protein in the high-micromolar range, we recently demonstrated that lycorine and its derivatives exhibit only weak DENV RdRp IC_50_ values (>100 µM), despite their potent antiviral EC_50_ values (<1 µM) [91]. This discrepancy represents an inhibitory concentration approximately 250 times higher than the cellular EC_50_ [90]. In contrast, for emetine, Yang et al. [50] clearly demonstrated direct inhibition of ZIKV NS5 RdRp with an IC_50_ of 0.121 µM, a concentration well within the pharmacologically relevant range, while cardiotoxicity was observed at concentrations 400–600-fold higher [68].

Although numerous compounds are listed as replication inhibitors in Table 2, Table 3, Table 4, Table 5 and Table 6, this does not imply that they act at the level of the RdRp. Direct enzymatic evidence exists only for lycorine against MERS-CoV and for emetine against ZIKV. For the remainder, the inhibition of replication reflects the outcome of time-of-addition or replicon experiments and does not distinguish among RdRp inhibition, interference with replication complex assembly, or translational effects on viral protein production.

### 3.3. Viral Protease Inhibition and Protease-Dependent Processing

Arbovirus genomes are translated into a single polyprotein that requires processing by host and viral proteases (Figure 2). Some evidence supports direct inhibition of protease by alkaloids. Li et al. [71] described lycorine as a main protease (M^pro^) inhibitor of SARS-CoV, using cytopathic effect-based screening, and the target was later confirmed experimentally for SARS-CoV-2 by Narayanan et al. [92] in a cell-based protease assay. To our knowledge, no equivalent data exist for flaviviral proteases (NS2B-NS3). Lycorine, like cherylline, did not inhibit a replication-deficient DENV replicon (NS5 catalytic mutant), which measures only cap-dependent translation of the input RNA [36]. This result demonstrates that lycorine’s anti-DENV activity requires active viral RNA replication.

Zou et al. (2009) reported resistance data that provide a solid link between lycorine and protease-dependent processing in WNV [38]. The V9M resistance mutation in the WNV viral genome, arising from continuous lycorine treatment, maps to the 2K peptide, a 23-amino-acid transmembrane segment between NS4A and NS4B. The cleavage at the NS4A-2K junction is performed by the viral NS2B-NS3 protease and is a prerequisite for the subsequent host signal peptidase (signalase) cleavage at the 2K-NS4B junction [93]. This sequential, protease-dependent processing of NS4A-2K-NS4B is essential for the membrane rearrangements that form the scaffold of the viral replication complex (Figure 1). The detected V9M mutation enhanced viral RNA replication ~100-fold in the presence of lycorine (but only 2–4-fold in its absence), possibly by altering 2K-mediated membrane reorganization or the efficiency of NS2B-NS3 cleavage at the NS4A-2K site. Equivalent genetic resistance maps have not yet been formally established for DENV or ZIKV. Therefore, while the weak in vitro inhibition of purified DENV/ZIKV NS5 RdRp supports an alternative target hypothesis, it remains to be determined whether the NS4A-2K-NS4B complex is targeted by lycorine across all flaviviruses, and this requires further virus-specific genetic validation.

Interestingly, Fikatas et al. [28] identified a novel series of indole alkaloid derivatives, structurally related to the ervatamine-silicine family, with a piperidine ring, that interfere with the replication complex through targeting NS4B, using genetically validated resistance mutations. Together with the specific targeting of the most clinically advanced DENV antiviral JNJ-1802, which blocks the NS3-NS4B protein–protein interaction [94], the lines of evidence converge on the central role of NS4B and its potency in association with NS3 in flavivirus replication. These findings position the NS4B-NS3 axis and the membrane-associated replication complex as a target warranting further investigation for alkaloid-based antivirals.

### 3.4. Other Viral Targets

Tylophorine and its derivatives exhibit nanomolar antiviral activity against coronaviruses by binding viral genomic/subgenomic RNAs and nucleocapsid proteins [95], but have not been tested against flaviviruses to our knowledge.

Overall, the most robust viral target assignments are currently that lycorine targets MERS-CoV RdRp but the NS4A-2K-NS4B membrane complex for WNV; emetine against ZIKV RdRp; and ervatamine-silicine-type indole derivatives target NS4B for DENV/ZIKV (resistance mutation). Strikingly, the two genetically validated targets in flaviviruses both implicate the NS4A-NS4B axis rather than NS5.

## 4. Host Factors as Targets Across Virus Families

As obligate intracellular pathogens, viruses rely on the host cell machinery to successfully complete their replication cycle and proliferate. While endogenous, these factors can be temporarily targeted to prevent viral replication and protect the host.

### 4.1. Ribosome Binding and Translational Stress: Old Evidence for an Emerging Antiviral Mechanism

The interaction of *Amaryllidaceae* alkaloids with the ribosome has been characterized at increasing resolution over five decades. Jimenez et al. (1976) demonstrated dose-dependent inhibition of protein synthesis by lycorine in mammalian cells [96]. Kukhanova et al. (1983) localized the target to the donor site of the peptidyltransferase center (PTC) of wheat-germ ribosomes [97]. Garreau de Loubresse et al. (2014) confirmed lycorine and narciclasine binding near the tRNA/mRNA interface by crystallography [98], and Pellegrino et al. (2018) solved the 3.1 Å structure of haemanthamine at the 80S ribosomal PTC A-site [99]. Consistent with a translational target, Li et al. (2021) used two independent CHIKV replicon systems to demonstrate that lycorine inhibits primarily viral translation [52]. Time-of-drug addition and SARS-CoV-2 replicon assays also suggest a post-entry step consistent with translation as the target of AAs [62]. Interestingly, despite a strong correlation between translation-inhibitory potency and EC_50_ against DENV and HCoV-OC43 [91], lycorine failed to inhibit a translation-only DENV replicon (NS5-deficient), demonstrating that active RNA replication is required for its antiviral effect [36]. Pleiotropism is also observed for berberine (Table 2) and possibly underscores that distinguishing translational from replicative effects remains technically challenging for compounds that may affect both processes [37].

Homoharringtonine (omacetaxine mepesuccinate), a cephalotaxane alkaloid approved for the treatment of chronic myeloid leukemia, binds to the same A-site cleft of the eukaryotic PTC [98], and the related harringtonine inhibits CHIKV replication by suppressing viral protein expression [30]. Homoharringtonine is also active against SARS-CoV-2 in vitro [68], although the mechanism was not specifically investigated in that study.

Narciclasine stands out for its steady EC_50_ (0.02 µM) across all four neglected flaviviruses tested (DENV, ZIKV, YFV, and JEV; Table 2 and Table 3) [39,42], a uniformity that suggests a host-directed mechanism, consistent with its known binding to the eukaryotic ribosomal PTC [98]. This potency is associated with a low SI (4–6), raising concerns about cytotoxicity. In addition, as narciclasine preferentially induces apoptosis in transformed cells while sparing normal cells such as fibroblasts [100], low SIs may underestimate the selectivity of narciclasine in primary cells. A similar caveat applies to pancratistatine, which was reported as cytotoxic and devoid of anti-SARS-CoV-2 activity in Vero-E6 cells [101], yet exhibits an exceptional SI (>23,000) against ZIKV, highlighting the strong influence of cell line and virus on selectivity outcomes [41]. A synthetic derivative, 7-hydroxyl-dihydronarciclasine, and trans-dihydronarciclasine derivatives displayed strong antiviral activity against SARS-CoV-2 (Table 5) and flaviviruses (Table 2), respectively, with enhanced SI (>100) [33,102]. Determining whether narciclasine inhibits a DENV translation-only replicon will help directly assess whether its anti-flaviviral activity operates through the ribosomal mechanism suggested by its structural data, or through a distinct pathway. Intriguingly, to our knowledge, narciclasine has not been tested against influenza or CHIKV, leaving its cross-family breadth undefined.

### 4.2. The Integrated Stress Response Following Ribosome Stalling

In addition to impairing protein synthesis, ribosomal inhibition has been suggested to trigger an integrated stress response (ISR) that may subsequently modulate the immune response. Independent antiviral observations suggest that translational stress could contribute to antiviral activity, though a definitive causal chain remains to be established. McNulty et al. (2023) reported ISR activation (PERK/eIF2α) by a truncated ring-A isocarbostyril [102] in HSV-1-infected brain organoids. PERK kinase upregulation leads to eIF2α phosphorylation and *ATF4* induction, the canonical ISR effector axis. A preprint [43] suggests that the binding of narciclasine and montanine to the ribosome PTC yields ribosome collision and triggers the ISR axis. While these preliminary findings position ISR activation as an intriguing candidate bridge between translational perturbation and the induction of an antiviral innate immune response, the precise causal relationship between alkaloid-dependent ribosome stalling, host ISR activation, and direct viral translation inhibition has yet to be validated. As preliminary evidence in RNA viruses, early upregulation of *ATF4* transcription was detected following treatment with antiviral *Amaryllidaceae* alkaloids in the context of HCoV-OC43 infection, correlating with an increase in *IFN-β* transcript levels (Table 5) [62]. Although ISR activation has been reported to intersect with innate antiviral signaling in some contexts, the pathway linking eIF2α phosphorylation to type I interferon production remains mechanistically undefined and distinct from the canonical *IRF3/IRF7*-dependent induction routes. Whether alkaloid-induced ribosome stalling leads to ISR activation that contributes to antiviral activity, or whether translational inhibition alone accounts for the observed effects, remains to be determined experimentally.

### 4.3. Host Innate Immune Signaling

Convergent lines of evidence suggest that alkaloids may modulate host innate immune signaling pathways. Cepharanthine was recently shown to reduce IL-6 production following DENV infection (Table 2) [86]. Giannone et al. demonstrated that berberine inhibits ZIKV-induced activation of both ERK1/2 and p38 MAPK in infected cells, and that the p38 inhibitor SB202190 phenocopied berberine’s antiviral effect (Table 2) [45]. Since p38 MAPK regulates inflammatory cytokines and modulates cap-dependent translation via *MAPKAPK2-*mediated phosphorylation of *eIF4E*, a pathway parallel to the eIF2α-centered ISR, berberine may engage the same translational-immune interface through a distinct upstream kinase. Berberine also impaired the formation of intracellular and extracellular infectious DENV particles (Table 2), thus involving late-stage assembly and host signaling. In a complementary observation, Huang et al. (2026) showed that lycorine restores the type I IFN response suppressed during infection with infectious bronchitis virus in chicken cells by upregulating MDA5 expression through the RIG-I-like receptor pathway rather than the ISR [103]. This suggests that some alkaloids may enhance innate antiviral immunity through multiple independent mechanisms, likely depending on the compound, virus, and cell type.

Of note, none of these observations demonstrate that immune modulation is the primary antiviral mechanism rather than a bystander effect. Translational inhibition alone, by reducing the burst of viral protein synthesis that positive-strand RNA viruses require, could account for much of the antiviral activity without requiring ISR signaling. Disentangling these possibilities will require experiments in which ISR or MAPK signaling is specifically blocked while monitoring viral replication in alkaloid-treated cells.

### 4.4. Additional Host-Targeting Mechanisms

At least three other host-directed antiviral pathways have been experimentally established for alkaloids. Lycorine derivatives downregulate the host chaperone Hsc70, destabilizing the HCV replication complex (Table 4) [59]. This represents a validated host-targeting pathway with potential transferability to DENV, where Hsc70 is also required for replication [104,105], though this has not been tested. Lycorine inhibits the synthesis of the nucleoporin Nup93, blocking nuclear export of influenza nucleoprotein [106], but there is no equivalent in flaviviruses (which replicate in the cytoplasm). Du et al. (2026) identified a novel mechanism for the lycorine derivative compound 7, stabilization of ZAP-S (zinc-finger antiviral protein, short isoform), which disrupts -1PRF, validated by in vivo data in hamster models (Table 5) [66]. This mechanism is conserved across SARS-CoV, SARS-CoV-2, and MERS-CoV.

Castanospermine and other iminosugars inhibit DENV infection at the level of secretion and viral infectivity by targeting host α-glucosidases, disrupting *N*-glycan processing and viral glycoprotein folding [46], a mechanism active against DENV, HCV, HIV, and influenza that has progressed furthest toward clinical application. For most alkaloid-arbovirus pairs, including the most potent (narciclasine, pancratistatine, amarbellisine), the molecular target remains unconfirmed. Resolving this deficit is an important challenge facing this field.

It is worth noting that DNA intercalation by isoquinoline alkaloids such as berberine and emetine is well characterized in the context of DNA viruses and protozoa [107,108]. For RNA viruses, however, the relevance of this mechanism is less clear. Pépin et al. (2017) demonstrated that DNA intercalating agents can activate cGAS-STING signaling through low-level host DNA damage, providing an alternative route to type I IFN induction [109]. Whether the alkaloids discussed here engage this pathway in addition to the ribosome-mediated ISR remains to be determined.

### 4.5. Structure-Activity Insights Across Alkaloid Scaffolds

**Figure 3:**
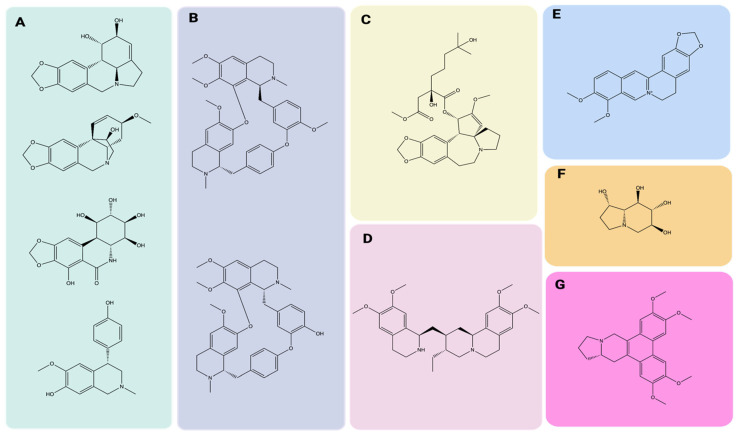
Chemical structures of representative antiviral alkaloid scaffolds discussed in this review, organized by botanical family. (**A**) *Amaryllidaceae* alkaloids: lycorine (pyrrolo[de]phenanthridine), haemanthamine (5,10b-ethanophenanthridine), pancratistatine (isocarbostyril), and cherylline (4-arylisoquinoline). (**B**) *Menispermaceae* bis-benzylisoquinolines: tetrandrine (top) and berbamine (bottom). (**C**) *Cephalotaxaceae*: homoharringtonine (cephalotaxane). (**D**) *Rubiaceae:* emetine (bis-tetrahydroisoquinoline). (**E**) *Berberidaceae:* berberine (protoberberine; quaternary ammonium). (**F**) *Fabaceae*: castanospermine (polyhydroxylated indolizidine iminosugar). (**G**) *Apocynaceae*: tylophorine (phenanthroindolizidine). These 11 compounds were selected to represent the structural diversity, cross-family antiviral breadth, and range of molecular targets identified in this review. All structures were drawn using ChemDraw 22.2.0 from their SMILES code.

Preliminary correlations between alkaloid scaffold architecture and the antiviral target class can be discerned from the growing body of literature (Figure 3A–G). *Amaryllidaceae* alkaloids (lycorine, haemanthamine, cherylline, pancratistatine), despite spanning different structural subtypes (pyrrolo[de]phenanthridine, 5,10b-ethanophenanthridine, 4-arylisoquinoline, and related isocarbostyril scaffold of pancratistatine), share a low molecular weight (~285–325 Da) and a fused multicyclic framework that restricts conformational flexibility (Figure 3A), and appear to preferentially target intracellular post-entry steps [36,62,89,91]. This suggests that their compact size and conformationally constrained polycyclic scaffolds favor binding to intracellular pockets, such as the ribosomal PTC or replication complexes, rather than to viral surface proteins or host membrane receptors. The convergent binding modes of haemanthamine and unrelated homoharringtonine [98,99] may point towards a pharmacophore of conformationally constrained polycyclic heterocycles whose core dimensions are compatible with the A-site cleft. While homoharringtonine (545 Da; Figure 3C) is considerably larger than haemanthamine (301 Da), its cephalotaxine core (~299 Da) that inserts into the PTC is comparable in size to the phenanthridine scaffolds.

In contrast to *Amaryllidaceae* alkaloids, large dimeric bis-BIQ (tetrandrine, berbamine, fangchinoline, cepharanthine; ~606–622 Da; Figure 3B) consistently inhibit viral entry across both *Coronaviridae* and *Flaviviridae*. Their large dimeric framework and dual protonatable nitrogen atoms appear to favor interaction with endolysosomal TRPML channels, disrupting endosomal trafficking [44,64,85].

Instead, berberine and emetine exhibit virus-dependent dual mechanisms, albeit through different structural logic. Berberine is a rigid, fully planar quaternary ammonium alkaloid (Figure 3E) whose flat aromatic surface facilitates DNA intercalation and direct interaction with protein targets. It blocks HCV entry through binding to the E2 glycoprotein [60] but acts post-entry against DENV and ZIKV by disrupting infectious particle formation and *p38 MAPK* signaling [45]. Emetine combines two aromatic domains connected by a flexible aliphatic linker (Figure 3D), which may enable adaptation to structurally diverse binding partners. This bis-tetrahydroisoquinoline potently inhibits ZIKV NS5 RdRp as well as blocks EBOV entry by disrupting lysosomal function [50]. Both compounds are documented DNA intercalators [107,108] via distinct binding modes, but whether this ability contributes to their antiviral activity remains undemonstrated. Interestingly, for the phenanthroindolizidine tylophorine (Figure 3G), another extended polyaromatic scaffold, nucleic acid binding appears directly linked to antiviral function: tylophorine binds viral RNA and nucleocapsid proteins at nanomolar concentrations [63], suggesting that polyaromatic alkaloids can exploit this property for antiviral activity when directed at viral rather than host targets.

Finally, castanospermine acts via host-targeting mechanisms independent of direct viral protein binding. The iminosugar is a polyhydroxylated indolizidine whose hydroxylation pattern closely mimics glucose, enabling competitive inhibition of ER-resident α-glucosidases and thereby disrupting N-glycan processing required for viral glycoprotein folding [46], a mechanism applicable to any enveloped virus that requires glycoprotein maturation (Figure 3F). Collectively, these observations suggest that alkaloid scaffold geometry, molecular weight, and charge distribution are informative predictors of antiviral target class (Figure 3), and that structure-guided scaffold selection may help prioritize candidates to address specific mechanistic gaps in arbovirus drug discovery.

### 4.6. Limitations

**Figure 4:**
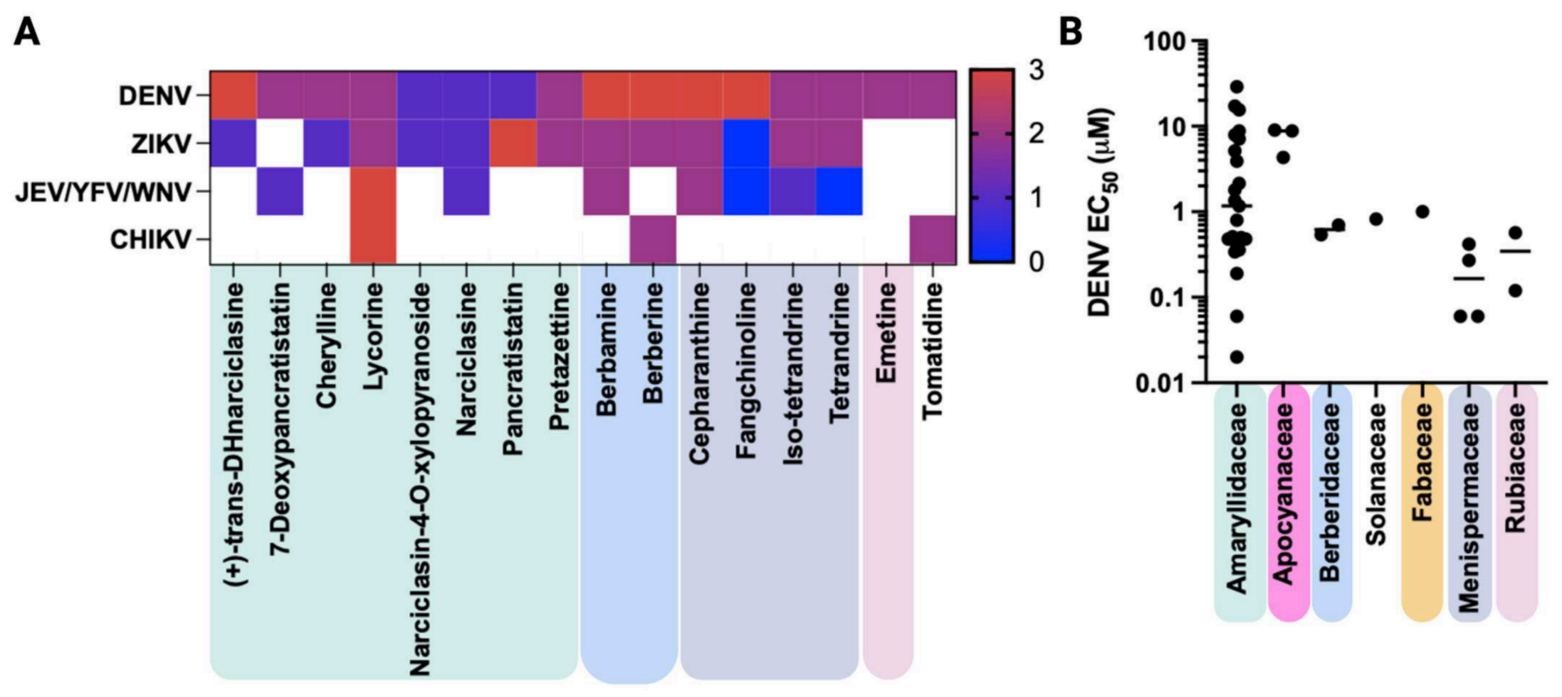
Antiviral alkaloid landscape across neglected arboviruses. (**A**) Heatmap of selectivity index (SI) strength for selected alkaloids compiled from the current literature. SI values were classified into four categories: 0 (SI < 5, blue; non-selective), 1 (SI 5–10, purple; marginal), 2 (SI 10–100, magenta; acceptable), and 3 (SI > 100, red; strong selectivity). White cells indicate untested compound-virus pairs. JEV, YFV, WNV, and AHFV were grouped due to limited individual data points. For compounds tested against multiple viruses within the same group, the highest SI was retained. Compounds are organized by botanical family: *Amaryllidaceae* (green), *Berberidaceae* (blue), *Menispermaceae* (lavender), and *Rubiaceae* (pink). (**B**) Distribution of EC_50_ values (µM, log_10_ scale) against DENV by plant family. Horizontal bars indicate medians. Figures were realized using GraphPad Prism v11.0.2.

This review has several limitations that should be acknowledged. First, as a critical narrative, the literature selection may be subject to bias. The predominance of *Amaryllidaceae* alkaloids in this survey partly reflects the research focus of the contributing groups but also the abundance of literature on this plant family (Figure 4). Crucially, it also mirrors a broader asymmetry in the field: compounds such as lycorine, narciclasine, and pancratistatine have historically received a significantly deeper level of structural and mechanistic characterization, particularly regarding host translation inhibition, compared to other classes like bis-BIQ, indoles, or quinolizidine alkaloids. Second, the heterogeneity of antiviral endpoints (EC_50_, IC_50_, MNTC), cell lines, and viral strains across the compiled studies precludes direct quantitative comparison of potencies between studies. As illustrated in Figure 4, antiviral testing remains heavily skewed toward DENV and ZIKV, with CHIKV as well as other arboviruses and non-*Amaryllidaceae* scaffolds largely unexplored despite mechanistic rationale for activity. The influence of cell line selection, particularly the use of transformed cells to which some alkaloids such as narciclasine are preferentially toxic, further complicates the assessment of therapeutic potential. Third, in vivo validation remains limited to a few compound-virus pairs, and pharmacokinetic, ADMET, and bioavailability data are lacking for most alkaloids discussed here. Known toxicity liabilities, including the cardiotoxicity of emetine and the inherent cytotoxicity of ribosomal inhibitors such as narciclasine, represent unresolved barriers to clinical translation. Systematic screening of bis-BIQ compounds against CHIKV and of under-represented plant families (*Apocynaceae*, *Fabaceae*, *Rutaceae*) against neglected arboviruses represents a tractable near-term priority.

## 5. Conclusions

This review compiled over 100 alkaloid-virus pairs across RNA virus families and identified five chemical scaffolds, isoquinoline (emetine, berberine), benzylisoquinoline, bis-benzylisoquinoline (tetrandrine, berbamine), pyrrolo[de]phenanthridine (lycorine) and isocarbostyril (narciclasine), as displaying cross-family antiviral activity. This analysis reveals both strengths and weaknesses in the field. The most robust mechanistic assignments come from well-characterized viral systems and are supported by enzymatic, structural, or genetic validation. In contrast, the widely cited lycorine-NS5 interaction for flaviviruses lacks enzymatic confirmation at pharmacologically relevant concentrations, and over half of all alkaloid-virus mechanisms remain entirely uncharacterized. Converging structural, biochemical, and transcriptomic evidence spanning five decades suggests that ribosome-mediated translational stress may constitute an additional, unifying mechanism for isoquinoline-derived alkaloids, including the *Amaryllidaceae* subgroup, but this hypothesis requires formal validation. Similarly, the reported modulation of innate immune pathways by several alkaloids may reflect secondary consequences of translational inhibition rather than independent antiviral mechanisms, and distinguishing these possibilities will require pathway-specific inhibition experiments. Addressing pharmacological limitations and the mechanistic deficit through unbiased target deconvolution, formal testing of the ribosome/ISR hypothesis, expanded taxonomic coverage, and in vivo validation will determine which plant-derived alkaloids can be safely translated into therapeutics for the neglected arboviruses that continue to threaten global health. To this end, chemical proteomics, genome-wide functional screens, and transcriptomic profiling would complement the mechanism-oriented analysis presented here by identifying targets that cannot be predicted from phenotypic assays alone, while network pharmacology and AI-driven structural optimization may accelerate the design of derivatives with improved selectivity.

## Figures and Tables

**Table 4:** Reported antiviral activity and mechanism of action of diverse alkaloid families against Hepatitis C virus.

	Alkaloid	EC_50_ (μM)	CC_50_ (μM)	SI	Target	Ref.
*Amaryllidaceae*	Lycorine	1.03 ± 0.12	9.9 ± 0.72	10	HSP70 (host-target mechanism)	[59]
*Berberidaceae*	Berberine	7.87 ± 1.10	82.75 ± 0.27	11	Entry (E2 glycoprotein binding)	[60]
*Piperaceae*	Piperine	* IC_50_ 52.18 ± 3.21	n.d.	n.d.	NS5B polymerase	[61]

Ref.: Reference. EC_50_: concentration of 50% inhibition of infection. *: represents IC50 values determined using a cell-based phenotypic assay. CC_50_: concentration of 50% cytotoxicity, SI: selectivity index (CC_50_/EC_50_ or IC_50_). ± standard deviation of the mean. n.d.: Not determined.

**Table 5:** Reported antiviral activity and mechanism of action of diverse alkaloid families against HCoV-OC43, SARS-CoV-2, and various coronaviruses.

	Alkaloid	EC_50_ (μM)	CC_50_ (μM)	SI	Target	Ref.
**HCoV-OC43**						
*Amaryllidaceae*	Lycorine	1.6 ± 0.4	34.9 ± 5.2	22	Replication or translation	[62]
	Haemanthamine	1.6 ± 0.7	49.2 ± 4.8	32		
	Haemanthidine	1.7 ± 0.6	36.7 ± 1.8	22		
	Crinamine	0.5 ± 0.1	25.2 ± 1.2	51		
	Amarbellisine	0.2 ± 0.1	12.1 ± 2.4	60		
	Cherylline	8.0 ± 1	>100	>13		
	Pancracine	2.6 ± 1.3	81.4 ± 11.8	31		
	Ungeremine	1.6 ± 0.3	6.6 ± 2.4	4		
	Clivimine	18.7 ± 9.9	>100	>5		
	Tazettine	21.6 ± 3.6	>100	>5		
	Obliquine	23.0 ± 0.6	>100	>4		
	11-hydroxyvittatine	23.3 ± 0.8	>100	>4		
*Apocynaceae*	Tylophorine	0.016 ± 5	>10,000	>610	Replication;	[63]
*Menispermaceae*	Tetrandrine	8.29	14.51	>40	Entry; Replication	[64]
	Fangchinoline	0.92	12.40	13		
	Cepharanthine	0.73	10.54	11		
*Rubiaceae*	Emetine	0.30	2.69	9	Replication	[65]
**SARS-CoV-2**						
*Amaryllidaceae*	Montanine	* IC_50_ 1.71	165	>96	Translation	[43]
*Amaryllidaceae (semi-synth.)*	Lycorine derivative compound 7	0.73	79.81	109	Translation (-1PRF)	[66]
*Apocynaceae*	Tylophorine	0.013	>10	>769	Replication: Transcription	[63]
*Berberidaceae*	Berbamine	IC_50_ 43.4	n.d.	n.d.	Entry: Postfusion core	[67]
*Cephalotaxaceae*	Homoharringtonine	2.55	59.75	23	n.e.	[68]
*Fabaceae*	Oxysophoridine	0.18	>40	>222	Replication	[69]
*Menispermaceae*	Tylophorine	0.0140	5.10	364	Replication; transcription	[63]
*Rubiaceae*	Emetine	0.46	56.46	123	Replication	[68,70]
**Other coronavirus**						
	**Alkaloid**	**EC_50_ (μM)**	**CC_50_ (μM)**	**SI**	**Target (Virus)**	**Ref.**
*Amaryllidaceae*	Lycorine	0.016 ± 0.001	14.98 ± 0.91	>936	Replication (SARS-CoV)	[71]
		* IC_50_ 1.021 ± 0.025	>50	49	Replication (SARS-CoV)	[72]
		* IC_50_ 1.406 ± 0.260	>50	36	Replication (RdRp) (MERS-CoV)	[72]
*Berberidaceae*	Berbamine	29.2 ± 7	n.d.	n.d.	Entry (MERS-CoV)	[73]
*Menispermaceae*	Tetrandrine	7.0 ± 0.8	n.d.	n.d.		
	Fangchinoline	1.7 ± 0.2	n.d.	n.d.		
*Rubiaceae*	Emetine	1.43	3.63	3	Replication (HCoV-NL63)	[65]

Ref.: Reference. EC_50_: concentration of 50% inhibition of infection. *: represents IC50 values determined using a cell-based phenotypic assay. CC_50_: concentration of 50% cytotoxicity. SI: selectivity index (CC_50_/EC_50_ or IC_50_). > indicates that the maximum nontoxic concentration tested did not reach the 50% cytotoxicity threshold (the corresponding SI values represent minimal calculated selectivity). ± standard deviation of the mean. -1PRF: -1 Programmed Ribosomal Frameshifting. RdRp: RNA-dependent RNA polymerase. n.d.: Not determined. HCoV-OC43: Human Coronavirus OC43. SARS-CoV: Severe Acute Respiratory Syndrome Coronavirus. SARS-CoV-2: Severe Acute Respiratory Syndrome Coronavirus-2. MERS-CoV: Middle East Respiratory Syndrome Coronavirus. HCoV-NL63: Human Coronavirus NL63.

**Table 6:** Reported antiviral activity and mechanism of action of diverse alkaloid families against diverse RNA viruses.

	Alkaloid	EC_50_ (μM)	CC_50_ (μM)	SI	Target	Ref.
**Other RNA Viruses**						
*Liliaceae*	Cyclopamine	* IC_50_ 0.46 to 0.82 in vitro	n.d.	n.d.	n.e./RSV	[74]
*Rubiaceae*	Emetine	* IC_50_ 10.2	>100	>9.8	Entry/EBOV	[50]
*Solanaceae*	Atropine	MNTC 5.53	n.d.	n.d.	n.e./HPIV-3	[75]
	Octopamine	MNTC 10.45	n.d.	n.d.		
**Lentivirus (HIV-1)**						
*Amaryllidaceae*	* Extract of *Crinum asiaticum* var. *japonicum*	ED_50_ 12.5 μg/mL	CD_50_ 200 μg/mL	16	Replication	[76]
*Ancistrocladaceae*	Michellamine D	3 (against HIV-RF)	n.d.	n.d.	n.e.	[77]
	Michellamine F	2 (against HIV-RF)	n.d.	n.d.		
*Apocynaceae*	18-Methoxycoronaridine	9.5 ± 3–12.8 ± 5	328	34.5–25.6	RT	[78]
*Fabaceae*	6-O-Butanoyl castanospermine (B-CAST)	* IC_50_ 0.19–0.58	n.d.	n.d.	Maturation	[79]
	Aloperine	1.75 ± 0.59	>86.2	>49.25	Entry	[80]
	Aloperine N-(1-butyl)-4-trifluoromethoxy-benzamide	0.69 ± 0.13	>42.1	>61.01		
*Lauraceae*	Hernandonine	^ IC_50_ 16.3	n.d.	n.d.	n.e.	[81]
	Laurolitsine	^ IC_50_ 7.7	n.d.	n.d.		
	7-oxohernangerine	^ IC_50_ 18.2	n.d.	n.d.		
	Lindechurine A	^ IC_50_ 21.1	n.d.	n.d.		
*Menispermaceae*	Fangchinoline	0.8–1.7	6.4–6.9	3.8–8.6	Maturation	[82]
*Rubiaceae*	Emetine	14.1	250	17.7	Entry; RT	[83]
*Rutaceae*	Buchapine	2.99	55.89	18.69	n.e.	[84]
	3-(3-methyl-2-butenyl)-4-[(3-methyl-2-butenyl) oxy]-2(1H)-quinolinone	3.8	71.17	18.72		
	4-(Isopentyloxy) quinolin-2-ol	3.9	114.67	29.47		

Ref.: Reference. EC_50_: concentration of 50% inhibition of infection. *: represents IC50 values determined using a cell-based phenotypic assay. ^: represents biochemical IC50, measured through a cell-free assay directly evaluating enzyme inhibition. CC_50_: concentration of 50% cytotoxicity. ED_50_: Effective Dose 50%. CD_50_: Cytotoxic Dose 50%. SI: selectivity index (CC_50_/EC_50_ or IC_50_) or (CD_50_/ED_50_). > indicates that the maximum nontoxic concentration tested did not reach the 50% cytotoxicity threshold; the corresponding SI values represent minimal calculated selectivity. ± standard deviation of the mean. n.d.: Not determined. n.e.: Not elucidated. MNTC: Maximum Non-Toxic Concentration, RSV: Respiratory Syncytial Virus, EBOV: Ebola Virus, HPIV-3: Human Parainfluenza Virus type 3, HIV-1: Human Immunodeficiency Virus type 1. RT: reverse transcriptase.

## Data Availability

No new data were created or analyzed in this study. Data sharing is not applicable to this article.
